# HIV-Related Knowledge and Perceptions by Academic Major: Implications for University Interventions

**DOI:** 10.3389/fpubh.2014.00018

**Published:** 2014-03-12

**Authors:** Matthew Lee Smith, Lisa L. La Place, Mindy Menn, Kelly L. Wilson

**Affiliations:** ^1^Department of Health Promotion and Behavior, College of Public Health, University of Georgia, Athens, GA, USA; ^2^Department of Social and Behavioral Health, School of Rural Public Health, Texas A&M Health Science Center, College Station, TX, USA; ^3^College of Health and Human Performance, University of Florida, Gainesville, FL, USA; ^4^Department of Health and Kinesiology, Texas A&M University, College Station, TX, USA

**Keywords:** HIV, AIDS, college students, academic major, perceived risk

## Abstract

Most universities offer human sexuality courses, although they are not required for graduation. While students in health-related majors may receive sexuality education in formal settings, majority of college students never receive formal sexual health or HIV/AIDS-related education, which may lead to elevated engagement in high-risk sexual behaviors. This study examines perceived knowledge about HIV/AIDS, perceived risk, and perceived consequences among college students by two distinct classifications of academic majors. Data were collected from 510 college students. Binary and multinomial logistic regressions were performed to compare HIV-related covariates by academic major category. Limited differences were observed by science, technology, engineering, and mathematics categorization. Relative to health and kinesiology majors, those who self-reported being “completely knowledgeable” about HIV were less likely to be physical sciences, mathematics, engineering, and business (PMEB) (OR = 0.41, *P* = 0.047) or education, humanities, and social sciences majors (OR = 0.25, *P* = 0.004). PMEB majors were less likely to report behavioral factors as a risk for contracting HIV (OR = 0.86, *P* = 0.004) and perceived acquiring HIV would be more detrimental to their quality of life (OR = 2.14, *P* = 0.012), but less detrimental to their mental well-being (OR = 0.58, *P* = 0.042). Findings can inform college-wide campaigns and interventions to raise HIV/AIDS awareness and improve college health.

## Introduction

The traditional college years represent a well-documented period of marked identity development and exploration both in and out of the classroom. As a facet of student development outside of the classroom, extracurricular experimentation with one’s sexual identity (with new sexual partners and with new sexual behaviors) is pervasive in the American college culture and spans across majors (1). Recently, many authors documented undergraduate students’ self-reported engagement in risky sexual behaviors such as engaging in sex while drunk, engaging in sex with multiple partners, and engaging in unprotected sex (2–6). These behaviors carry inherent and varied risks of HIV transmission, yet the American College Health Association’s National College Health Assessment (ACHA-NCHA) revealed a majority of students surveyed had never been tested for HIV (*n* = 17,166; 73.4%) and were not interested in receiving sexually transmitted disease prevention information from their institution (*n* = 14,823; 63.7%).

Within the classroom and the broader academic realm of higher education, students and university personnel are concerned with a student’s academic field of study or “major” since the selection, progression, and completion of a degree program are hallmarks of the university experience (5, 7–11). The major is prevalent in the American higher education system and provides benefits for students and for institutions. For students, the major provides a predetermined plan of study; varying in flexibility between departments, fields, and institutions. The major is the framework for prescribing coursework selected by scholars in the field, and approved by the degree-granting institution. Selecting a college major signals the beginning of a journey toward developing competence regarding a specific subject and narrowing career options to those that meet the students’ interests and abilities honed through major coursework. For institutions, majors provide a consistent basis to classify, group, and divide students, faculty, physical, and financial resources.

Though every institution’s leaders have varied freedom to group majors into colleges, departments, or schools for organizational purposes, an emerging method of demarcating and dichotomizing majors is the use of the science, technology, engineering, and mathematics (STEM) categorization. Though a universal definition of STEM or list of STEM majors does not exist (12), the United States Immigration and Customs Enforcement (an agency operating within the Department of Homeland Security) publishes a STEM degree program list that it uses to classify college majors as STEM or Non-STEM. This designation is then used to grant or deny foreign-born college graduates optional practical training work permits.

Within the last decade, initiatives from the National Science Foundation, the White House, National Academies, and the United States Department of Education increased funding for programs to increase students’ interest, enrollment, and completion of STEM degrees; further cementing the dichotomy of STEM and non-STEM majors. As a natural result of this increased funding, dichotomization, and attention, numerous authors have compared STEM and non-STEM majors regarding enrollment, persistence, and graduation rates (13–17). Conversely, no identified researchers have explored the relationship between students’ majors and perceptions of HIV; therefore the purposes of this study were to: (1) identify participant’s perceived knowledge about HIV/AIDS, perceived risk, and perceived consequences among college students; and (2) examine how these factors differ by academic major. Recognizing that students in certain fields of study are exposed to more health protective information than students in other disciplines, the authors hypothesized that health and kinesiology (HK) majors would self-report higher levels of knowledge regarding HIV/AIDS and have more accurate perceptions of HIV-related risk factors and consequences than students in other majors.

## Materials and Methods

The “Finding Roots: Exploring Your Family History” study examined college students’ knowledge and perceptions regarding family health history and chronic diseases (i.e., HIV/AIDS, cancer, heart disease, diabetes, cystic fibrosis, and being overweight or obese) (18–21). The survey was developed based on the health belief model (22) and consisted of 60 items; included Likert-type scales, checklists, and close-ended response formats. The 60 items addressed investigators’ primary interest areas including: communication about family history and disease among family members, perceptions of severity and incidence regarding several common chronic diseases, perceived causes of diseases, perceived consequences of getting the disease, and a battery of items pertaining to the participants’ sociodemographics. Additional information about the survey instrument is available upon request to the corresponding author.

The survey was disseminated online through course listserv without attached course credit or extra credit and 703 students voluntarily responded. Institutional Review Board approval was received for this study, which oversees the ethical and professional standards of research involving human subjects. Of the 703 survey participants, those who indicated they had been diagnosed with HIV (*n* = 5), knew someone diagnosed with HIV (*n* = 69), and were enrolled in graduate school (*n* = 66) were omitted from analyses to reduce bias concerning HIV/AIDS-related knowledge level and risk perception. Further, participants who did not report their academic major were excluded from this study (*n* = 54). Thus, the resulting analytic sample consisted of 510 undergraduate students.

### Measures

#### Dependent variable: primary and secondary majors

Two classification levels of college students’ self-reported academic majors were used as dependent variables for this study. Academic majors were primarily dichotomized into STEM and non-STEM categories. Then, the authors segmented all reported academic majors into four secondary classification groups based on common nature, associated skill sets, and professional philosophies. The four secondary academic major groups included: (1) physical sciences, mathematics, engineering, and business (PMEB); (2) education, humanities, and social sciences (EHS); (3) biological sciences (BS); and (4) HK. Table 1 presents the primary and secondary academic major categories used for analyses in this study.

**Table 1:** **Academic majors presented by STEM status and secondary academic major groups**.

Science, technology, engineering, and mathematics (STEM)	Non-STEM majors	Physical sciences, mathematics, engineering, business (PMEB)	Education, humanities, and social sciences (EHS)	Biological sciences (BS)	Health and kinesiology (HK)
Aerospace engineering	Accounting	Accounting	Agricultural leadership, education, and communications	Animal science	Health and kinesiology
Animal science	Agricultural economics	Aerospace engineering	Anthropology	Biochemistry/biophysics	
Biochemistry/biophysics	Agricultural leadership, education, and communications	Agricultural economics	Communication	Biological and agricultural engineering	
Biological and agricultural engineering	Anthropology	Architecture	Distance learning	Biology	
Biology	Architecture	Civil engineering	Educational administration	Biomedical engineering	
Biomedical engineering	Communication	Chemical engineering	Educational psychology	Biomedical science program	
Biomedical science program	Economics	Computer science	English	Entomology	
Chemical engineering	Educational administration	Construction science	Hispanic studies	Forest science	
Civil engineering	Educational psychology	Economics	History	Geography	
Computer science	English	Electrical and computer engineering	Human resource development	Geology and geophysics	
Construction science	Finance	Engineering technology and industrial distribution	Performance studies	Horticultural science	
Electrical and computer engineering	Forest science	Finance	Philosophy	Nutrition and food science	
Engineering technology and industrial distribution	Geography	Industrial and systems engineering	Political science	Oceanography	
Entomology	Health and kinesiology	Information and operations management	Psychology	Plant pathology and microbiology	
Geology and geophysics	Hispanic studies	Landscape architecture and urban planning	Recreation, park, and tourism sciences	Poultry science	
Horticultural science	History	Management	Sociology	Veterinary physiology and pharmacology	
Industrial and systems engineering	Human resource development	Marketing	Teaching, learning, and culture	Wildlife and fisheries sciences	
Information and operations management	Landscape architecture and urban planning	Mathematics			
Mathematics	Management	Mechanical engineering			
Mechanical engineering	Marketing	Nuclear engineering			
Nuclear engineering	Nutrition and food science	Ocean engineering			
Ocean engineering	Performance studies	Petroleum engineering			
Oceanography	Philosophy	Physics			
Petroleum engineering	Political science				
Physics	Psychology				
Plant pathology and microbiology	Recreation, park, and tourism sciences				
Poultry science	Sociology				
Veterinary physiology and pharmacology	Teaching, learning, and culture				
Wildlife and fisheries sciences					

#### HIV/AIDS knowledge and risk perceptions

Variables used to identify college students’ perceptions about HIV/AIDS included: self-rated HIV/AIDS knowledge (i.e., if the participant had no/low knowledge, was somewhat knowledgeable, was completely knowledgeable); and susceptibility of contracting HIV in their lifetime (i.e., if the participant perceived to have no chance of contracting HIV, be unlikely to contract HIV, be likely to contract HIV).

#### Factors influencing HIV transmission

Participants ranked their beliefs about the extent to which five factor types attributed to an individual contracting HIV (i.e., behavioral, social, genetic, environmental, and spiritual). Participants were asked, “On a scale from 1 to 10, how much do (blank) factors influence your likelihood to develop HIV?” Higher scores indicated the participant believed that factor had more influence on an individual contracting HIV. Responses for each of these five items were recorded independently and treated as continuous variables.

#### Consequences of HIV

Participants ranked their beliefs about the extent to which six aspects of their life would be impacted if they were to contract HIV (i.e., quality of life, physical well-being, mental well-being, social life, emotional well-being, spiritual well-being). Participants were asked, “On a scale from 1 to 7, how detrimental would having HIV be to your ______?” Higher scores indicated the participant believed having HIV would be more detrimental to that aspect of their life. Responses for each of these six items were recorded independently and treated as continuous variables.

#### Personal characteristics

Personal characteristics of the participants included: age group (i.e., 18, 19, 20 years, between ages 21 and 24 years); sex; and race/ethnicity (i.e., non-Hispanic white, racial/ethnic minority).

### Data analysis

All statistical analyses were performed using SPSS (version 17). Frequencies were calculated for all categorical study variables, which were initially examined in relationship to the primary and secondary academic major categories. Pearson’s chi-square tests were performed to assess the independence between the dependent variable and categorized independent variables. Independent sample *t*-tests and one-way ANOVA was used to evaluate the mean differences among academic major categories for continuous variables. Bonferroni *post hoc* analyses were performed to identify the origins of significant mean differences. Binary logistic regression was used to identify personal characteristics, self-reported HIV knowledge, factors believed to influence HIV transmission, and perceived consequences of HIV associated with participants’ academic majors by STEM status (i.e., the non-STEM category served as the referent group). Multinomial logistic regression was used to identify associations among these factors with participants’ secondary academic major groups (i.e., the HK category served as the referent group).

## Results

### Sample characteristics

Participants’ personal characteristics, self-reported HIV-related knowledge, and perceived risk for contracting HIV are presented in Table 2. Of these 510 undergraduate college students, 41.6% were enrolled in majors categorized as STEM majors and in regard to the secondary academic major groups, 28.2% were PMEB majors, 22.6% were EHS majors, 24.3% were BS majors, and 24.9% were HK majors. The majority of participants were 20–24 years of age (67.9%), female (62.4%), and non-Hispanic white (74.3%). Approximately one-quarter of participants self-reported having no/low levels of HIV/AIDS-related knowledge, compared to 55.7% who reported being somewhat knowledgeable, and 19.0% who reported being completely knowledgeable. The majority of students reported having no chance of contracting HIV in their lifetime (57.3%) and approximately 10% of participants reported they were likely to contract HIV in their lifetime.

**Table 2:** **Sample characteristics by STEM major status and secondary academic major groups**.

	Total (*n* = 510) (%)	STEM status				Secondary academic major groups					
		Non-STEM (*n* = 298) (%)	STEM (*n* = 212) (%)	χ^2^	*P*	PMEB (*n* = 160) (%)	EHS (*n* = 115) (%)	BS (*n* = 108) (%)	HK (*n* = 127) (%)	χ^2^	*P*
**Age group (in years)**				18.43	<0.001					38.46	<0.001
18	10.4	6.7	15.6			13.8	6.1	13.9	7.1		
19	21.8	19.5	25.0			30.6	18.3	17.6	17.3		
20	31.6	37.2	23.6			26.3	24.3	29.6	46.5		
21–24	36.3	36.6	35.8			29.4	51.3	38.9	29.1		
**Sex**				79.86	<0.001					114.81	<0.001
Male	37.6	21.5	60.4			70.0	24.3	33.3	12.6		
Female	62.4	78.5	39.6			30.0	75.7	66.7	87.4		
**Race**				2.41	0.121					3.50	0.321
Non-Hispanic white	74.3	76.8	70.8			73.1	70.4	73.1	80.3		
Racial/ethnic minority	25.7	23.2	29.2			26.9	29.6	26.9	19.7		
**Perceived HIV/AIDS knowledge**				5.00	0.082					24.78	<0.001
No/low knowledge	25.3	25.8	24.5			30.0	29.6	20.4	19.7		
Somewhat knowledgeable	55.7	58.4	51.9			53.1	61.7	47.2	60.6		
Completely knowledgeable	19.0	15.8	23.6			16.9	8.7	32.4	19.7		
**Likelihood of contracting HIV: lifetime**				3.60	0.166					6.16	0.406
No chance	57.3	60.7	52.4			55.6	64.3	51.9	57.5		
Unlikely	32.7	29.9	36.8			35.0	24.3	36.1	34.6		
Likely	10.0	9.4	10.8			9.4	11.3	12.0	7.9		

When comparing characteristics by the academic major categories, statistically significant differences were observed. A significantly larger proportion of STEM majors were in younger age groups (χ^2^ = 18.43, *P* < 0.001); whereas, a significantly smaller proportion of STEM majors were female (χ^2^ = 79.86, *P* < 0.001) in comparison to their non-STEM counterparts. In terms of secondary academic major groups, larger proportions of PMEB and BS majors were in younger age groups relative to EHS and HK majors (χ^2^ = 38.46, *P* < 0.001). A significantly smaller proportion of PMEB majors (30.0%) were female compared to those in other majors (ranging from 66.7 to 87.4%) (χ^2^ = 114.81, *P* < 0.001). Significantly larger proportions of PMEB (30.0%) and EHS (29.6%) majors reported having no/low levels of HIV/AIDS-related knowledge, whereas a significantly larger proportion of BS (32.4%) majors reported being completely knowledgeable (χ^2^ = 24.78, *P* < 0.001).

### HIV/AIDS-related influences and consequences

Table 3 contains comparisons by academic major of the extent to which participants’ believed certain factors influence contracting HIV. On average (using a scale from 1 to 10), participants believed behavioral factors have the most influence on contracting HIV (*M* = 7.49 ± 3.68), followed by social factors (*M* = 6.70 ± 3.70), genetic factors (*M* = 4.18 ± 3.99), environmental factors (*M* = 3.87 ± 3.52), and spiritual factors (*M* = 1.72 ± 2.85). No significant mean differences were observed by STEM status. However, *post hoc* examinations of mean differences by secondary academic major group revealed HK participants believed behavioral factors had significantly more influence on a person contracting HIV relative to their PMEB counterparts (*f*  = 3.85, *P* = 0.010). HK participants also believed environmental factors had significantly more influence on a person contracting HIV relative to their EHS counterparts (*f*  = 2.78, *P* = 0.041).

**Table 3:** **HIV/AIDS-related beliefs by STEM status and secondary academic major groups**.

	Total (*n* = 510)	Non-STEM (*n* = 298)	STEM (*n* = 212)	*t*	*P*	PMEB (*n* = 160)	EHS (*n* = 115)	BS (*n* = 108)	HK (*n* = 127)	*f*	*P*
**BELIEF THAT GETTING HIV IS INFLUENCED BY**											
Behavioral factors	7.49 (±3.68)	7.58 (±3.65)	7.35 (±3.71)	0.70	0.486	6.85 (±3.96)	7.25 (±3.81)	7.81 (±3.58)	8.24 (±3.09)	3.85	0.010
Social factors	6.70 (±3.70)	6.64 (±3.79)	6.78 (±3.57)	−0.41	0.680	6.58 (±3.74)	6.18 (±4.06)	6.72 (±3.65)	7.30 (±3.27)	1.94	0.123
Genetic factors	4.18 (±3.99)	4.15 (±3.95)	4.22 (±4.04)	−0.18	0.861	3.98 (±4.07)	3.98 (±3.99)	4.24 (±3.95)	4.57 (±3.91)	0.64	0.589
Environmental factors	3.87 (±3.52)	3.91 (±3.61)	3.82 (±3.39)	0.31	0.757	3.69 (±3.41)	3.49 (±3.58)	3.65 (±3.39)	4.64 (±3.63)	2.78	0.041
Spiritual factors	1.72 (±2.85)	1.77 (±2.90)	1.66 (±2.79)	0.42	0.674	1.59 (±2.73)	1.68 (±2.82)	1.59 (±2.73)	2.04 (±3.13)	0.71	0.544
**CONTRACTING HIV WOULD BE DETRIMENTAL TO MY**											
Quality of life	6.20 (±1.22)	6.23 (±1.18)	6.16 (±1.29)	0.68	0.499	6.09 (±1.30)	6.21 (±1.32)	6.31 (±1.15)	6.25 (±1.10)	0.75	0.526
Physical well-being	6.17 (±1.27)	6.24 (±1.18)	6.08 (±1.38)	1.42	0.156	6.00 (±1.41)	6.17 (±1.32)	6.31 (±1.17)	6.28 (±1.08)	1.78	0.151
Social life	5.99 (±1.37)	6.07 (±1.29)	5.88 (±1.48)	1.50	0.134	5.72 (±1.57)	6.01 (±1.39)	6.14 (±1.26)	6.19 (±1.13)	3.45	0.017
Mental well-being	5.92 (±1.42)	6.05 (±1.27)	5.74 (±1.61)	2.40	0.017	5.61 (±1.63)	5.97 (±1.42)	6.03 (±1.37)	6.18 (±1.10)	4.36	0.005
Emotional well-being	5.89 (±1.47)	6.01 (±1.33)	5.73 (±1.64)	2.11	0.036	5.54 (±1.73)	5.97 (±1.44)	6.10 (±1.28)	6.09 (±1.20)	4.86	0.002
Spiritual well-being	4.52 (±2.16)	4.76 (±2.05)	4.17 (±2.26)	3.03	0.003	4.06 (±2.20)	4.73 (±2.08)	4.48 (±2.23)	4.93 (±2.02)	4.45	0.004

Table 3 also contains comparisons, by academic major, of the extent to which participants’ perceived contracting HIV would be detrimental to various aspects of their life. On average (using a scale from 1 to 7), participants perceived contracting HIV would be most detrimental to their quality of life (*M* = 6.20 ± 1.22), followed by their physical well-being (*M* = 6.17 ± 1.27), social life (*M* = 5.99 ± 1.37), mental well-being (*M* = 5.92 ± 1.42), emotional well-being (*M* = 5.89 ± 1.47), and spiritual well-being (*M* = 4.52 ± 2.16). When compared by STEM status, STEM majors perceived contracting HIV would be significantly less detrimental to their mental well-being (*t* = 2.40, *P* = 0.017), emotional well-being (*t* = 2.11, *P* = 0.036), and spiritual well-being (*t* = 3.03, *P* = 0.003) compared to their non-STEM counterparts. *Post hoc* examinations of mean differences by secondary academic major group revealed HK participants perceived contracting HIV would be significantly more detrimental to their social lives (*f*  = 3.45, *P* = 0.017), mental well-being (*f*  = 4.36, *P* = 0.005), and spiritual well-being (*f*  = 4.45, *P* = 0.004) compared to their PMEB counterparts, respectively. HK and BS participants perceived contracting HIV would be more detrimental to their emotional well-being relative to PMEB majors, respectively (*f*  = 4.86, *P* = 0.002).

### HIV/AIDS-related factors associated with academic major

Table 4 displays the results of the binary logistic regression analysis that examined sociodemographics and HIV-related factors associated with academic majors by STEM status (non-STEM served as the referent group). Participants who were female (OR = 0.17, *P* < 0.001) and those who believed contracting HIV would be detrimental to their spiritual well-being (OR = 0.87, *P* = 0.015) were statistically significantly less likely to be STEM majors.

**Table 4:** **Factors associated with STEM academic majors**.

			95% CI	
	OR	*P*	Lower	Upper
Age: 18 years	1.00	–	–	–
Age: 19 years	3.13	0.001	1.55	6.33
Age: 20 years	1.40	0.222	0.82	2.41
Age: 21–24 years	0.73	0.210	0.44	1.20
Male	1.00	–	–	–
Female	0.17	<0.001	0.11	0.27
Non-Hispanic white	1.00	–	–	–
Racial/ethnic minority	1.30	0.285	0.81	2.09
HIV knowledge: no/low	1.00	–	–	–
HIV knowledge: somewhat	0.69	0.238	0.37	1.28
HIV knowledge: completely	0.71	0.205	0.42	1.21
HIV lifetime risk: no chance	1.00	–	–	–
HIV lifetime risk: unlikely	0.81	0.545	0.40	1.62
HIV lifetime risk: likely	1.31	0.466	0.63	2.73
Behavioral factors as HIV risk	0.96	0.297	0.90	1.03
Social factors as HIV risk	1.04	0.285	0.97	1.12
Genetic factors as HIV risk	1.03	0.256	0.98	1.10
Environmental factors as HIV risk	0.98	0.632	0.91	1.06
Spiritual factors as HIV risk	0.97	0.483	0.90	1.05
Detrimental: quality of life	1.33	0.164	0.89	2.00
Detrimental: physical well-being	0.96	0.858	0.65	1.44
Detrimental: social life	1.02	0.900	0.78	1.33
Detrimental: mental well-being	0.84	0.295	0.60	1.17
Detrimental: emotional well-being	1.09	0.587	0.80	1.48
Detrimental: spiritual well-being	0.87	0.015	0.78	0.97

*Referent group: non-stem majors*.

Table 5 displays the results of the multinomial logistic regression analysis that examined sociodemographics and HIV-related factors associated with secondary academic major group (HK majors served as the referent group). The first model compared PMEB majors to HK majors. Relative to HK majors, participants who were 20 years of age (OR = 0.24, *P* = 0.006), 20–24 years of age (OR = 0.35, *P* = 0.047), female (OR = 0.05, *P* < 0.001), and completely knowledgeable about HIV/AIDS (OR = 0.41, *P* = 0.047) were statistically significantly less likely to be PMEB majors. Compared to HK majors, PMEB majors were statistically significantly less likely to believe behavioral factors influenced a person contracting HIV (OR = 0.86, *P* = 0.004). Compared to HK majors, those who believed contracting HIV would be detrimental to their quality of life were statistically significantly more likely to be STEM majors (OR = 2.14, *P* = 0.012); whereas, those who believed contracting HIV would be detrimental to their mental well-being were statistically significantly less likely to be STEM majors (OR = 0.58, *P* = 0.042).

**Table 5:** **Factors associated with secondary academic major groups**.

	PMEB				EHS				BS			
			95% CI				95% CI				95% CI	
	OR	*P*	Lower	Upper	OR	*P*	Lower	Upper	OR	*P*	Lower	Upper
Age: 21–24 years	0.35	0.047	0.13	0.99	2.05	0.212	0.66	6.31	0.57	0.259	0.21	1.52
Age: 20 years	0.24	0.006	0.09	0.66	0.58	0.353	0.19	1.82	0.29	0.013	0.11	0.77
Age: 19 years	0.71	0.523	0.24	2.05	1.03	0.962	0.31	3.44	0.49	0.193	0.17	1.44
Age: 18 years	1.00	–	–	–	1.00	–	–	–	1.00	–	–	–
Female	0.05	<0.001	0.03	0.11	0.46	0.036	0.22	0.95	0.31	0.001	0.15	0.63
Male	1.00	–	–	–	1.00	–	–	–	1.00	–	–	–
Racial/ethnic minority	1.05	0.883	0.53	2.08	1.45	0.266	0.75	2.79	1.38	0.344	0.71	2.71
Non-Hispanic white	1.00	–	–	–	1.00	–	–	–	1.00	–	–	–
HIV knowledge: completely	0.41	0.047	0.17	0.99	0.25	0.004	0.09	0.64	1.38	0.444	0.61	3.16
HIV knowledge: somewhat	0.60	0.138	0.30	1.18	0.64	0.171	0.33	1.22	0.72	0.372	0.36	1.47
HIV knowledge: no/low	1.00	–	–	–	1.00	–	–	–	1.00	–	–	–
HIV lifetime risk: likely	1.19	0.747	0.42	3.33	1.29	0.606	0.49	3.45	2.12	0.131	0.80	5.65
HIV lifetime risk: unlikely	1.20	0.570	0.64	2.23	0.69	0.245	0.37	1.29	1.43	0.243	0.78	2.62
HIV lifetime risk: no chance	1.00	–	–	–	1.00	–	–	–	1.00	–	–	–
Behavioral factors as HIV risk	0.86	0.004	0.78	0.95	0.94	0.203	0.85	1.04	0.94	0.228	0.85	1.04
Social factors as HIV risk	1.05	0.401	0.94	1.16	0.98	0.663	0.88	1.08	1.01	0.790	0.92	1.12
Genetic factors as HIV risk	1.03	0.490	0.95	1.12	1.03	0.512	0.95	1.11	1.03	0.395	0.96	1.12
Environmental factors as HIV risk	0.95	0.336	0.86	1.05	0.93	0.169	0.84	1.03	0.91	0.070	0.83	1.01
Spiritual factors as HIV risk	0.94	0.266	0.84	1.05	0.99	0.865	0.89	1.10	0.98	0.746	0.88	1.09
Detrimental: quality of life	2.14	0.012	1.18	3.88	1.65	0.099	0.91	2.99	1.21	0.556	0.65	2.25
Detrimental: physical well-being	1.15	0.650	0.63	2.09	0.96	0.907	0.52	1.78	1.41	0.274	0.76	2.59
Detrimental: social life	0.80	0.260	0.54	1.18	0.87	0.523	0.58	1.32	0.91	0.662	0.60	1.39
Detrimental: mental well-being	0.58	0.042	0.34	0.98	0.68	0.178	0.39	1.19	0.58	0.051	0.34	1.00
Detrimental: emotional well-being	1.15	0.506	0.76	1.75	1.17	0.476	0.76	1.81	1.45	0.117	0.91	2.29
Detrimental: spiritual well-being	0.87	0.084	0.75	1.02	1.00	0.955	0.85	1.16	0.89	0.105	0.76	1.03

*Referent group: HK majors*.

The second model compared EHS majors to HK majors. Relative to HK majors, female participants (OR = 0.46, *P* = 0.036) and participants who self-reported to be “completely knowledgeable” about HIV/AIDS (OR = 0.25, *P* = 0.004) were significantly less likely to be EHS majors than HK majors. The third model compared BS majors to HK majors. Relative to HK majors, participants who were 20 years of age (OR = 0.29, *P* = 0.013) and female (OR = 0.31, *P* = 0.001) were significantly less likely to be BS majors.

## Discussion

As previously noted, analyses of relationships between students’ majors and numerous characteristics and patterns are prevalent in published research. However, there is a paucity of research examining relationships between students’ majors and their health behaviors, health knowledge, and perceptions regarding chronic diseases; especially examining relationships of STEM and non-STEM majors with health concepts. To date, perceptions about HIV/AIDS have yet to be examined in relation to academic major. Although college students report high levels of knowledge about the HIV/AIDS epidemic, previous studies have shown many misconceptions about this condition persisting among undergraduate students (23–26). To complement and augment the prior research, the authors of this study utilized an instrument based on the health belief model to examine how HIV/AIDS-related knowledge, perceived risk, and perceived consequences differed by academic major among students enrolled in a Texas university. The findings confirmed the existence of variation pertaining to HIV-related factors by academic major within this cohort.

While high school students are strongly encouraged to select STEM majors upon entry into college to achieve academic success and subsequent vocational opportunities, their exposure to formal health-related education and associated content is inherently limited. As such, when comparing factors related to HIV/AIDS-related knowledge, perceived risk, and perceived consequences, it seems plausible that STEM majors would be significantly different from non-STEM majors in a variety of ways. However, findings from the current study revealed only few significant differences based on STEM status, thus warranting our reclassification into four secondary academic major groups. This reclassification enabled us to compare HIV/AIDS-related factors by more practically grouped academic categories, based on curricula similarities and exposure to health-related content.

As hypothesized, the responses of HK majors significantly differed from other majors, especially PMEB majors. While further research is needed to fully understand the nuances of these differences, we offer possible interpretations of these findings below. HK majors reported being more knowledgeable about HIV/AIDS compared to their counterparts enrolled in other majors. Perceived differences in HIV/AIDS-related knowledge by major may reflect the nature of the HK major, which comprise an array of health-related course requirements and electives (e.g., community health, human sexuality, health disparities, and epidemiology). By tailoring the design and delivery of course content, instructors may present HIV/AIDS-related content pertaining to prevalence and incidence rates, modes of transmission, and risk and protective factors.

In terms of factors influencing HIV/AIDS transmission, PMEB majors perceived behavioral factors to have less influence on contracting HIV/AIDS, when compared to HK majors. One possible interpretation for this finding may be that PMEB majors perceive their future employment opportunities to have less risk for being exposed to HIV/AIDS, unlike their HK counterparts who frequently enter allied health professions (e.g., nursing) where exposure to blood-borne pathogens may exist as an inherent occupational risk. This finding may also indicate that PMEB majors have not been exposed to HIV/AIDS-related education and are either naive about modes of HIV transmission or embrace historical stereotypes that HIV/AIDS is only an issue among those of lower socioeconomic status, intravenous drug users, or men who have sex with men.

Health and kinesiology majors reported contracting HIV would be more detrimental to their lives (e.g., mental well-being, social life, emotional well-being, and spiritual well-being) relative to their counterparts from other majors. This finding begs to question whether HK majors truly know and understand more about the realities of living with HIV/AIDS and thus have more accurate perceptions of the risks and ramifications of the disease. The realization of the negative impacts of living with HIV may be associated with being educated about the balance among the dimensions of health (e.g., physical, mental, emotional, social, spiritual, vocational) (27, 28), which are necessary to be considered healthy (i.e., beyond the mere absence of physical illness) (29). Conversely, considering HIV is increasingly considered a “chronic condition” (30–32), it seems feasible to believe HK majors would also be aware of the various mechanisms that exist to alleviate burdens associated with HIV/AIDS. Further, HK students, compared to PMEB students, may be more familiar with the well-documented relationships between spirituality and overall health status among individuals with chronic conditions (33, 34).

This study is not without limitations. The questionnaire used to collect data from participants was not specifically designed for the purposes of this study, thus it did not include items measuring actual HIV-related risk (e.g., being sexually active, using condoms or other barrier methods, using intravenous drugs, working in an environment with an elevated risk of needle-sticks), knowledge about modes of HIV transmission, or clinical consequences of getting HIV/AIDS (e.g., opportunistic infection, mortality). While the close-ended and Likert-type scales included in the instrument were useful to initially assess participants’ perceptions of HIV-related risk, severity, and consequences, future studies should include open-ended items or a qualitative inquiry (e.g., interviews, focus groups) to further investigate the sources of HIV knowledge as well as information/drivers about perceived risk and consequences. Such a qualitative study would be beneficial to professionals when developing educational and behavior change interventions in university settings. Additionally, the instrument did not include items regarding participants’ K-12 sexuality education experiences or if they ever took a human sexuality course at the college level. Further, the majority of sample participants were over 19 years of age, thus their HIV/AIDS-related knowledge, views, and beliefs may have differed from their younger counterparts who recently enrolled in the university setting. Data were self-reported and drawn from a convenience sample, thus biases associated with social desirability and self-selection may exist. Further, data were cross-sectional in nature and collected from students at one college campus, thus findings may not be representative of other college student populations and should not be widely generalized beyond this study sample. To build upon these findings, future studies should be conducted across a larger number of college campuses of varying sizes. Such studies could include colleges that widely offer and have high enrollment in human sexuality courses and examine the level of education (or exposure to content) needed to influence students’ sexual behavior and HIV/AIDS-related perceptions.

Although this study did not collect information pertaining to students’ enrollment in human sexuality courses, further research is needed to determine if exposure to basic health education content has potential to increase HIV/AIDS-related knowledge and perceptions about causal factors and ramifications. From the college administration perspective, strategies can be employed to increase all students’ exposure to sexuality content despite academic major. For example, to increase college students’ exposure to HIV/AIDS-related content in formal education settings, college curricula may be modified to require human sexuality as a mandated core course, or component of a core course, for graduation. Such formal education requirements may take the form of freshman seminars or one-credit courses. However, informal sexuality education mechanisms are often available on college campuses to complement sexuality education efforts offered in formal settings. Examples of informal education may utilize the reach of student health organizations to provide peer sexuality education in a variety of settings (e.g., dormitories, fraternities/sororities, other university-sanctioned organizations) to positively impact student knowledge and perceptions about HIV/AIDS-related issues (35). Peer education for health promotion interventions has the potential to help reduce risky sexual behaviors among college students by increasing their knowledge and self-efficacy for condom-use, refusing sexual intercourse and negotiating safer practices with a partner (36).

The observed correlation between exposure to sexuality content and perceived knowledge about sexual risk-taking behaviors may have implications that transcend formal curricula modification and informal peer sexuality education. Within this discussion, we must also consider sexuality education through a larger lens that encompasses experiences during primary and secondary schooling. When a college student enrolls in and attends their first semester on a university campus, they bring a vast range of preconceived attitudes and beliefs as well as a predefined level of knowledge about sex and HIV/AIDS. Therefore, depending on the level of sexuality education received prior to college enrollment, students’ sexual health competency and ability to protect themselves from contracting sexually transmitted infections (or preventing unplanned pregnancies) inherently differs. If students are not required to receive sexuality education through their college curricula, the expectation for these individuals to protect themselves and their classmates from negative sexuality-related ramifications can only be dictated from other sources including family, friends, or the media, which may not relay accurate or credible information (37–39). To complement educational efforts, evidence suggests the effectiveness of group-based behavioral interventions to encourage protective behaviors, reduce risk-taking activities, and prevent disease transmission (40). Such interventions have potential for integration in existing healthy sexuality initiatives on college campuses and can be facilitated by university health clinics or other campus-affiliated entities.

## Conclusion

The content, timing, and delivery mechanisms of sex education and HIV education are hotly debated topics within the United States’ K-12 system yet largely ignored or downplayed in the higher education system. These discrepancies are largely attributed to variation in regulations from state-to-state and district-to-district, which dictate and restrict sexuality education curricula and sexual health content delivered in the classroom (41). These inconsistencies have potential to deprive young adults from obtaining the necessary sexual health information needed to protect themselves from STI and foster engagement in risky behavior once students discover the freedoms inherent to many college experiences. The reasons associated with differences in knowledge and perceptions about HIV/AIDS risk and consequences across undergraduate majors are relatively unexplored and deserve more attention. If we gain a better understanding of these issues, we can better inform the formal college/university core curriculum and informal on-campus interventions related to HIV/AIDS, to increase knowledge and skills among college students who likely have not and will not be exposed to sexuality education in their academic coursework. Without a focus on such activities, communication about this topic may never occur. Overall, undergraduates have a wide variety of knowledge and skills about HIV/AIDS. This paper recommends further study to explore what type of education and support students with various majors need in order to understand the risk and prevention of HIV/AIDS. Students enter and progress through their undergraduate education with highly varied levels of knowledge related to general sexual health and consequences associated with risky sexual behavior (42, 43). Human sexuality is rarely mandated for graduation on college campuses; however, certain majors offer such courses through required courses or electives (44). Thus, students in health-focused academic majors have an added opportunity for exposure to human sexuality education or HIV/AIDS prevention. Additionally, sexuality topics may be incorporated in courses with a different primary focus of the course (e.g., program planning, interventions, program evaluation, epidemiology, community health).

Consequently, some fields of study may never include HIV/AIDS-related education in a formal setting. Thus, these students may have less knowledge about risk factors, disease transmission, as well as health and social ramifications of certain sexual behaviors. To public health professionals who have participated in professional preparation that included healthy sexuality and safe sex, these behaviors may seem common sense, but for a young adult who has not encountered the information may not comprehend their risk levels. The ramifications of human sexuality research cross academic discipline lines yet the human sexuality discussions are still relegated to health departments and student health care clinics.

## Author Contributions

Matthew Lee Smith designed the study, collected the data, performed data analyses, conceptualized the study, and wrote portions of the manuscript. Lisa L. La Place, Mindy Menn, and Kelly L. Wilson conceptualized the study and wrote portions of the manuscript.

## Conflict of Interest Statement

The authors declare that the research was conducted in the absence of any commercial or financial relationships that could be construed as a potential conflict of interest.
